# A Two-Step Approach to Tune the Micro and Nanoscale Morphology of Porous Niobium Oxide to Promote Osteointegration

**DOI:** 10.3390/ma15020473

**Published:** 2022-01-08

**Authors:** Paolo Canepa, Giuseppe Firpo, Elena Gatta, Roberto Spotorno, Paolo Giannoni, Rodolfo Quarto, Maurizio Canepa, Ornella Cavalleri

**Affiliations:** 1 Dipartimento di Fisica and OPTMATLAB, Università di Genova, Via Dodecaneso 33, 16146 Genova, Italy; canepap@fisica.unige.it (P.C.); canepa@fisica.unige.it (M.C.); 2 Dipartimento di Fisica and NANOMED, Università di Genova, Via Dodecaneso 33, 16146 Genova, Italy; firpo@fisica.unige.it; 3 Dipartimento di Fisica, Università di Genova, Via Dodecaneso 33, 16146 Genova, Italy; gatta@fisica.unige.it; 4 Dipartimento di Chimica e Chimica Industriale, Università di Genova, Via Dodecaneso 31, 16146 Genova, Italy; roberto.spotorno@unige.it; 5 Dipartimento di Medicina Sperimentale, Università di Genova, Via Leon Battista Alberti 2, 16132 Genova, Italy; paolo.giannoni@unige.it (P.G.); rodolfo.quarto@unige.it (R.Q.); 6 U.O. Oncologia Cellulare, IRCCS Ospedale Policlinico San Martino, 16132 Genova, Italy; 7 INFN, Sezione di Genova, Via Dodecaneso 33, 16146 Genova, Italy

**Keywords:** niobium, ASD, etching, SEM, AFM, XPS, Ca/P, osteoblast

## Abstract

We present a two-step surface modification process to tailor the micro and nano morphology of niobium oxide layers. Niobium was firstly anodized in spark regime in a Ca- and P-containing solution and subsequently treated by acid etching. The effects of anodizing time and applied potential on the surface morphology is investigated with SEM and AFM, complemented by XPS compositional analysis. Anodizing with a limiting potential of 250 V results in the fast growth of oxide layers with a homogeneous distribution of micro-sized pores. Cracks are, however, observed on 250 V grown layers. Limiting the anodizing potential to 200 V slows down the oxide growth, increasing the anodizing time needed to achieve a uniform pore coverage but produces fracture-free oxide layers. The surface nano morphology is further tuned by a subsequent acid etching process that leads to the formation of nano-sized pits on the anodically grown oxide surface. In vitro tests show that the etching-induced nanostructure effectively promotes cell adhesion and spreading onto the niobium oxide surface.

## 1. Introduction

A major concern in the design of orthopedic and dental implants is the integration of the implant in the bone tissue. It is well known that cellular processes such as adhesion, growth, and differentiation are largely influenced by the physicochemical properties of the surface. In particular, surface morphology, chemical composition, or electric charge can deeply influence the cell response [1,2,3]. Therefore, large efforts have been focused on the surface modification of implant materials. Alongside the most widely used titanium and its alloys (in particular the medical titanium alloy Ti-6Al-4V) [4,5,6], many studies have been conducted on other bio-metals (aluminum, tantalum, magnesium, zirconium, niobium, and their alloys), employing anodic oxidation to grow porous oxide layers enriched in osteoconductive ionic inclusions [7,8,9,10,11,12,13,14,15,16,17,18]. Pointing to niobium, attention has been focused on the oxide enrichment in Ca and P compounds for osteointegration purposes using various electrolytes: a porous oxide layer structure has been reported, with morphology and composition depending on electrolyte, applied potential, limiting current and process time [19,20,21,22,23,24]. Together with macro roughness, which can help to mimic the bone trabecular structure, micro/nano roughness has been reported to play a key role to stimulate osteoblast adhesion and proliferation [2,3,25,26,27,28,29,30]. The submicrometer morphology can also help in preventing the adhesion of bacteria, limiting adhesion points on the material surface and causing mechanical rupture of bacterial cell membranes [31,32,33,34,35].

While the pore size of anodic oxide can be tuned by changing the applied anodizing conditions, it is difficult to tailor both the micro and the nanoscale roughness simultaneously. Indeed, when anodizing produces micropores, their borders usually present rather flat areas. Aiming to control both the micro and nano roughness, we propose a two-step surface modification process, combining anodizing with chemical etching. Anodizing is firstly employed to obtain oxide layers with micro-sized pores and chemical etching is subsequently used to add nano roughness to the porous surface. Etching processes have been widely exploited to modify the surface morphology of titanium and its alloys: an increase in surface roughness with the formation of micro/submicro pits has been reported upon acid etching at different acid concentration, etching temperature and time [36,37,38,39,40,41]. Surface morphology modifications with an increase in surface roughness and the formation of porous submicrometer structures has been reported upon chemical treatment in alkaline solutions at room [42,43] or higher temperature [41,44,45]. A hierarchical morphology exhibiting micro-valleys and nanostructures has been obtained combining acid etching and hydrothermal alkali treatment [25].

In this work, we applied adouble surface modification process on pure niobium sheets. A first anodizing step was performed in a solution containing calcium and phosphorous salts, with limiting potentials of 200 V and 250 V in galvanostatic and galvanostatic/potentiostatic mixed regimes. SEM and AFM images were acquired to evaluate the sample surface morphology and roughness. Surface ion inclusions were evaluated by XPS analysis. A second surface modification step was performed on anodized samples using etching in a H_2_O_2_/HCl solution. The effectiveness of the etching treatment on cell adhesion was investigated with in vitro cell culture tests using human osteosarcoma MG-63 cells.

## 2. Materials and Methods

### 2.1. Substrate Preparation

Commercially pure 2 mm thick niobium sheets (kindly provided by Dr. Musenich, INFN Genova) were mechanically cut to 1 cm × 1 cm squares. Before anodizing, substrates were polished using a Struers Labopol-5 machine equipped with rotating SiC papers P1000 ÷ P2500 grain size (Fepa-P scale). After polishing, samples were ultrasonically rinsed in Milli-Q water (resistivity 18 MΩ·cm, Millipore, Merck KGaA, Darmstadt, Germany), ethanol (99.8% purity, Sigma-Aldrich, Merck KGaA, Darmstadt, Germany), acetone (99.5% purity, Sigma-Aldrich, Merck KGaA, Darmstadt, Germany), and finally rinsed again in Milli-Q water. Samples were dried at room temperature under a gentle N2 flow.

### 2.2. Anodic Oxidation

A two-electrode cell using a Pt grid as counter-electrode was used to anodize samples. An Agilent N5751A (Keysight) power supply controlled through a LabVIEW (National Instruments) procedure was used for anodizing. During anodizing, the solution was stirred at a rate of about 500 rpm. Anodizing was carried out using the following solution: 0.2 M calcium acetate Ca(OOCCH_3_)_2_, 0.1 M phosphoric acid H_3_PO_4_, and 0.2 M di-sodium ethylenediamine tetraacetate Na_2_(EDTA). The as-prepared solution had pH 4. Limiting anodizing potentials of 200 V and 250 V were explored, using a pure galvanostatic regime or a galvanostatic/potentiostatic mixed regime. In the first case, samples were anodized in a galvanostatic regime (current density 350 mA/cm^2^), stopping the process as soon as the limiting potential was reached (in the following, we will refer to these samples as Nb200V and Nb250V). In the second case, at the end of the galvanostatic regime, the anodizing process was carried on in potentiostatic regime at the limiting attained potential for a total anodizing time of 60 s, 300 s, or 30 min (in the following, we will refer to these samples as Nb200V60s, Nb200V300s, and Nb200V30min for anodizing at 200V; Nb250V60s and Nb250V300s for anodizing at 250 V). See SI (Figure S1) for current density/potential vs. time curves.

### 2.3. Etching

After anodizing, samples were etched in a mixture of 9 M H_2_O_2_ and 0.3 M HCl at 75 °C for 30 min. Etched samples were rinsed in Milli-Q water and dried under a N_2_ flux.

### 2.4. Surface Characterization Methods

#### 2.4.1. Surface Analysis (SEM)

A Crossbeam 1540 XB High-Resolution Field Emission Scanning Electron Microscope (HR-FE-SEM, Carl Zeiss, Germany) with a resolution of 1.1 nm equipped with in-lens and Everhart-Thornley (E-T) detectors was used for surface characterization. Images were acquired with a working distance of about 4 mm, an electron energy of 20 keV, and an aperture of 30 µm. The E-T secondary electron detector was employed to enhance topographic contrast. For images at high magnification, in-lens detector was used to reveal nanometer details.

#### 2.4.2. Roughness Analysis (AFM)

Sample morphology was analyzed with a Bruker Nanoscope V system Atomic Force Microscope (AFM) operated in tapping mode. Si cantilevers (OMCL-AC160TS, Olympus) with nominal resonance frequency of ~300 kHz and nominal tip radius of ~7 nm were used. Data were analyzed with Nanoscope v3.15 software. Surface roughness was evaluated through the analysis of the AFM images using the roughness R_a_:

$$R_{a}=\frac{1}{n}\sum_{i=1}^{n}\left|z_{i}-\bar{z}\right|$$

where $\bar{z}$ is the mean height, $z_{i}$ is the height of the single point, and n is the number of points of the image. The relative uncertainty on the average R_a_ values is of 10%.

#### 2.4.3. Chemical Analysis (XPS)

A 5600 Multi-Technique apparatus (PHI, U.S.A.) equipped with an Al-monochromatized X-ray source (1486.6 eV) and an Omni Focus III MultiChannel detector was used for chemical surface analysis. To avoid sample charging, a neutralizer was used (low energy electron gun). A pass energy of 23.5 eV was used to acquire high resolution spectra. The binding energy scale was calibrated by setting the C1s component of adventitious carbon at 284.8 eV. Spectra were analyzed with CasaXPS software; after a Shirley background subtraction, 30% gaussian Voigt functions and spin-orbit splittings of 3.55 eV for Ca2p and 0.86 eV for P2p were used for spectra deconvolution.

### 2.5. Cell Culture

Human osteosarcoma MG-63 cells were used for the cell culture experiments. The cell line was available in the lab and originally purchased from Banca Cellule-Interlab Cell Line collection (ICLC) IRCCS Ospedale Policlinico San Martino, Genova-16132, Italy. Cells were cultured in Dulbecco’s Modified Eagle Medium (DMEM) supplemented with 10% fetal bovine serum, 1 mM glutamine, and 1% penicillin/streptomycin (Merck, KGaA, Darmstadt, Germany). The cell cultures were maintained at 37 °C in a humidified 5% CO_2_/95% air atmosphere. For passaging, cells were detached with trypsin/EDTA and subsequently replated. For the experiments, the cells were seeded at a density of 10^4^ cells/ 24-well plate and cultured for 48 h on the substrate of interest. Prior to use, the substrates were sterilized by immersion in absolute ethanol, rinsing in pure water and autoclave sterilization. Immediately prior to cell seeding, substrates were further sterilized under ultraviolet light for 10 min. For SEM analysis, the samples were further treated by fixing the MG-63 cells with a solution of 3% glutaraldehyde in PBS for 24 h at 4° C. Subsequently, samples were dehydrated in 30%, 50%, 70%, 90%, 95%, and 100% ethanol for 10 min each step. Pure ethanol was replaced with HMDS and samples were air dried.

## 3. Results

### 3.1. Anodizing

**Figure 1:**
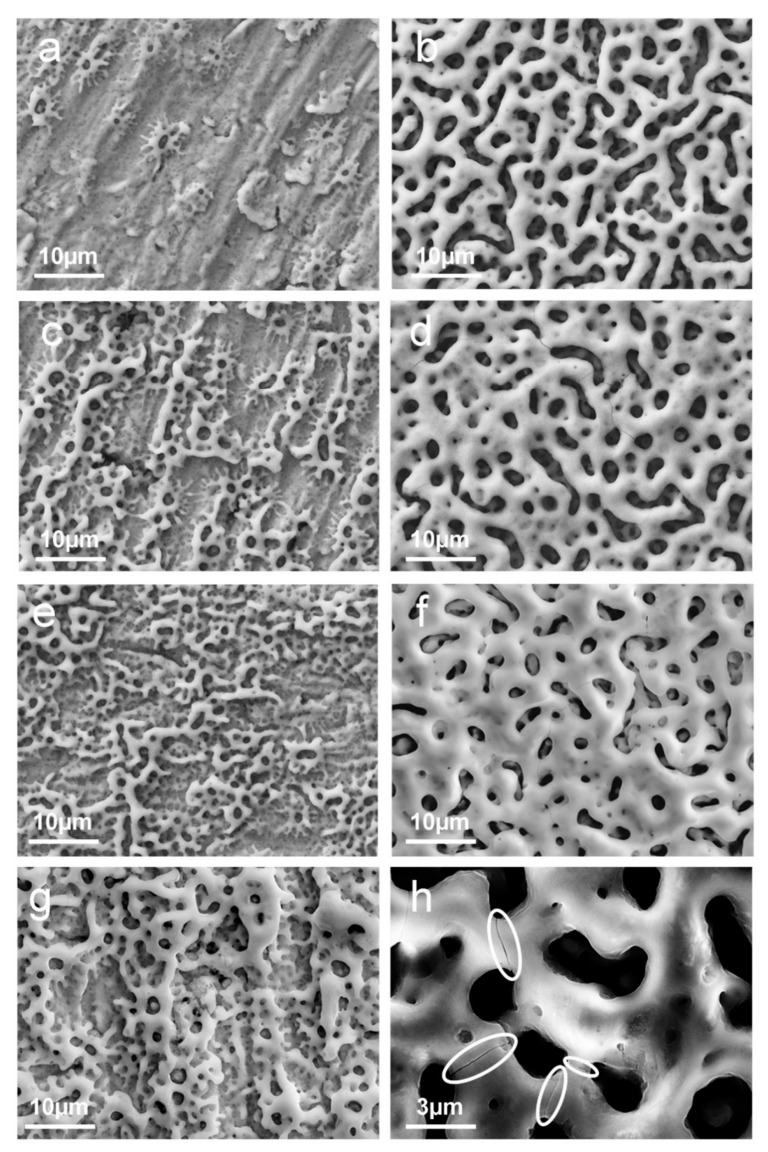
Representative top view SEM images (E–T detector) of anodized sample at limiting potential of 200 V (left column) and 250V (right column) in different conditions: for (**a**,**b**) galvanostatic process, (**c**,**d**) galvanostaic/potentiostatic (60 s), (**e**,**f**) galvanostaic/potentiostatic (300 s), and (**g**) galvanostaic/potentiostatic (30 min). (**h**) High magnification of the fractures (circled) observed in samples anodized in pure galvanostatic regime at 250 V (10 s).

We performed anodization experiments with limiting potentials of 200 V and 250 V in pure galvanostatic and mixed galvanostatic/potentiostatic regimes to evaluate the best conditions for the growth of uniform Ca- and P-enriched porous oxide layers. Representative SEM images of niobium anodized at 200 V and 250 V for different anodizing times are reported in Figure 1. Figure 1a,b show the effect of anodization in pure galvanostatic regime (with a current density of 350 mA/cm^2^), while images of samples anodized for longer times in a mixed galvanostatic/potentiostatic regime are reported in Figure 1c–g. To highlight the effects of anodizing on sample morphology, a typical SEM image of a Nb sheet before anodizing is reported for comparison in SI, Figure S2.

The observation of sparks in all the anodizing conditions employed in this work indicates that the oxide growth occurs in the Anodic Spark Deposition (ASD) regime, as confirmed by the presence of pores. The comparison between left column images (limiting potential 200 V) and right column images (limiting potential 250 V) indicates that the anodizing potential has a large effect on the oxide morphology. This is particularly evident from the comparison of samples anodized in pure galvanostatic conditions (Figure 1a,b). Anodizing with 200 V limiting potential produces an uneven distribution of almost round pores, with polishing scratches still visible on the surface. Conversely, after anodization with a limiting potential of 250 V the surface is uniformly covered by a porous oxide layer, with the presence of elongated pores likely resulting from the coalescence of adjacent pores. It is worth noting that pure galvanostatic anodization is roughly three times longer in the second case (~10 s for sample in Figure 1b vs. ~2 s for sample in Figure 1a; see current density/potential vs. time curves in SI, Figure S2). However, 10 s anodizing at 250 V (Figure 1b) produces a more uniform porous layer than a 30 s anodizing at 200 V (Figure 1c). Therefore, the effect of the attained potential on the surface morphology is definitely larger than the effect of the anodizing time.

Anodizing at 250 V is therefore very effective in rapidly producing a uniform porous oxide coverage, and increasing the anodizing time leads to an overall thickening of the pore walls. However, a closer analysis shows that samples, regardless of anodizing time, exhibit fractures in the oxide layer (Figure 1h, circles), likely due to the mechanical breakdown [46] which occurs during oxide growth in the ASD regime [19,47,48,49]. As we reported for a closely related system [23], the presence of fractures worsens the passivation properties of the oxide layer. Therefore, to avoid crack formation, we focused on anodization at 200 V, increasing the anodization time moving from pure galvanostatic to mixed galvanostatic/potentiostatic regime (Figure 1a,c,e,g). A significantly slower process occurs when the limiting potential is decreased from 250 V to 200 V.

Isolated micropores, formed in galvanostatic conditions (Figure 1a), gradually develop over the surface until an almost uniform porous structure is observed, after 30 min anodization (Figure 1g). 

**Figure 2:**
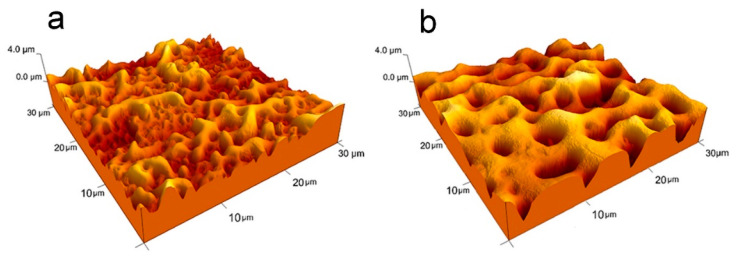
3D visualization of typical 30 µm × 30 µm (4 µm z-scale) AFM images of (**a**) Nb200V60s and (**b**) Nb250V60s samples.

In parallel to SEM, we used AFM imaging to obtain information on the vertical excursion of the oxide surface. As an example, representative images of Nb samples anodized in a mixed galvanostatic/potentiostatic regime at 200 V and 250 V are reported in Figure 2. From the analysis of the AFM images, we could evaluate the surface roughness R_a_ (calculated on a 100 µm × 100 µm scan size area).

As reported in Table 1, roughness values are in the range of hundreds of nm, with an increasing trend with increasing anodizing time for both 200 V and 250 V limiting potential. Larger Ra values are observed on samples anodized at higher potential. The leveling of Ra for long anodization at 250 V (Nb250V300s) is reasonably related to the thickening of the pore borders, with the reduction in the empty volumes at the surface.

The increase in roughness with the applied potential is in agreement with reports in literature on similar systems [19,20,22], even though a direct comparison of roughness values is not straightforward since they depend on the specific anodizing conditions (electrolytes, current density, and anodizing time) and measuring methods (e.g., AFM, stylus or optical profilometry).

**Figure 3:**
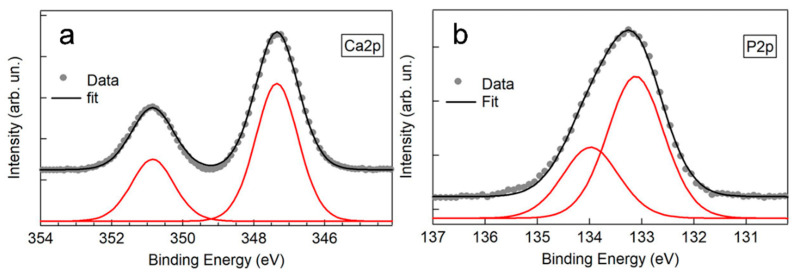
Representative XPS high-resolution spectra of (**a**) Ca2p and (**b**) P2p core regions of anodized niobium.

The comparative morphological analysis reported above indicates that long anodization at 200 V produces the best results among the explored conditions since it leads to the formation of rather homogeneous fracture-free porous layers. From the compositional point of view, XPS measurements indicate the presence of Ca and P in all the samples, a sought-after result in view of osteointegration purposes. Typical Ca2p and P2p core level regions are reported in Figure 3. In both cases, the signal can be reproduced by a single doublet, with the main 2p3/2 component at (347.3 ± 0.2) eV for Ca, and (133.1 ± 0.2) eV for P. A quantitative analysis of Ca2p, P2p, and Nb3d signal intensities indicates that Ca/P and (Ca + P)/Nb ratios increase with the anodizing time for samples anodized at 200 V while they are roughly independent on the anodizing time for samples anodized at 250 V. Ca/P of about 0.7 and (Ca + P)/Nb of about 1.2 are measured for Nb200V30min samples, comparable with 0.8 and 1.2 for Nb250V. The combined morphological and compositional analysis identifies long anodization at 200 V as the best anodizing condition which produces a Ca- and P-enriched, fracture-free and uniform porous oxide layers.

### 3.2. Post-Anodizing Treatments

**Figure 4:**
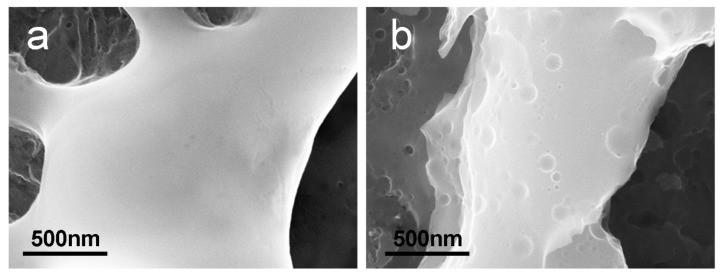
High resolution SEM images of post-anodizing etching effects: (**a**) a pore border of a typical anodized sample; (**b**) nano-sized pits on a pore border after etching.

As observed in the previous section, anodizing allows tailoring of both the chemical composition and morphology of Nb surfaces. A high surface roughness with the presence of pores, aiming to mimic the bone trabecular structure, is a sought-after feature for osteointegration purposes. At the same time, nano roughness can influence the first steps of osteoblast attachment and differentiation. Indeed, a hierarchical micro-nanostructuring of the surface topography has been reported to improve the osteogenic activity of titanium oxide [27,50,51]. Anodizing produces porous surfaces but, as can be observed by high magnification SEM images (Figure 4a), the surface of the pore borders is quite smooth. To achieve micro- and nano-structuring we applied an etching process to anodized samples. Chemical etching is a widely exploited method to modify surface morphology. Alkaline metal treatments [44], hydrogen peroxide [29], and acid etching processes [30,39,40,52] have been applied on pure titanium and titanium alloys to modify surface roughness and the effectiveness of the process in terms of bioactivity has been assessed by in vitro cell culturing, or by monitoring hydroxyapatite deposition after soaking in simulated body fluid (SBF).

We etched anodized niobium in a mixture of 9 M H_2_O_2_ and 0.3 M HCl at 75° for 30 min. The effects of the post-anodizing etching can be observed in the SEM image reported in Figure 4b. As previously reported in Section 2.4.1, to highlight the presence of nano-sized structures, SEM images were acquired using an in-lens detector. While the overall porous structure was almost unaffected by the etching process, nano-sized pits, with diameters in the nm range from 10 nm to 100 nm, were present on the borders of the pre-formed micropores. The double anodizing/etching process was therefore successful in tailoring both the micro and nano morphology of niobium oxide. Double surface modification processes have been reported in literature to produce oxide layers with tailored properties [19,49,53]. Two-step anodization in Ca- and P-containing electrolytes was reported to produce hydroxyapatite coatings on niobium [19]. Niobium oxide layers with enhanced corrosion resistance were obtained by plasma electrolytic oxidation coupled with electrophoretic deposition [49]. Recently, Ossowska et al. proposed a combined thermal and electrochemical oxidation process to produce crystalline oxide layers with an upper nanotubular oxide coating [53]. Here, we combine anodization and etching to add nano roughness to micro-sized pores of the oxide surface.

### 3.3. In Vitro Tests

**Figure 5:**
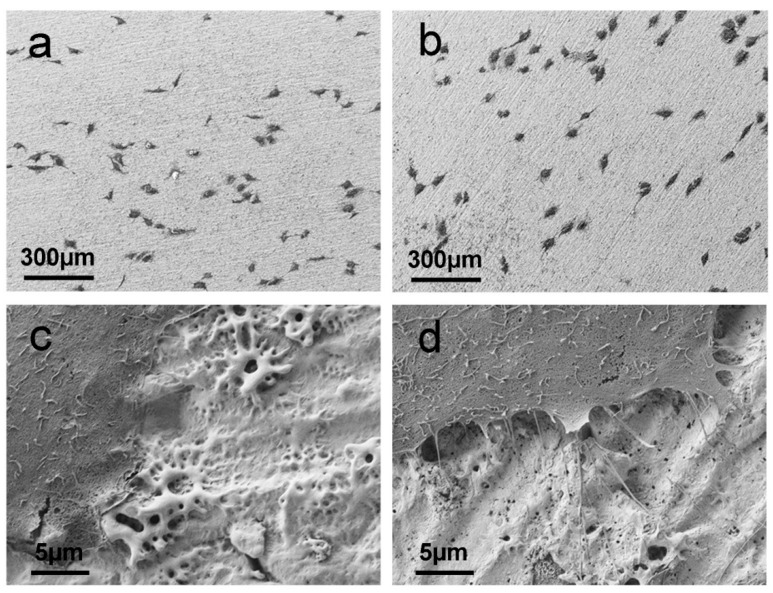
SEM images at (**a**,**b**) low and (**c**,**d**) high magnification of MG-63 osteosarcoma cells after 48 h incubation on (**a**,**c**) Nb200V and (**b**,**d**) Nb200V + etching.

To assess the influence of surface nanomorphology on cell adhesion and spreading, we performed in vitro tests using a MG-63 osteosarcoma cell line. SEM micrographs of MG-63 cells cultured for 48 h on anodized niobium and anodized/etched niobium are reported in Figure 5. At low magnification, a similar behavior in terms of cell density and morphology is observed for cells cultured on anodized Nb (Figure 5a) and on anodized and subsequently etched samples (Figure 5b). In both cases, cells seem to be attached and well adherent to the surface, with similar cell shape and distribution on the two substrates. However, high resolution SEM images show some differences between the two samples. In particular, cells incubated on anodized and etched Nb (Figure 5d) show well pronounced cytoplasmatic projections and well developed filopodia which are almost absent in the case of cells incubated on anodized niobium (Figure 5c).

Several reports investigated the cell response to structured transition metals or alloys. An enhanced osteoblast response has been reported for micro/nano-textured titanium obtained via etching followed by anodization [51]. An increased cell spreading induced by surface nano-features was reported also by Lauria et al. [29] who investigated cell differentiation on Ti45Nb samples treated by hydrogen peroxide oxidative etching. Micro and nanotextured Ti6Al4V surfaces obtained by H_2_SO_4_/H_2_O_2_ etching [30] were reported to promote osteoblast proliferation while inhibiting fibroblast growth. Nanotubular oxide layers grown on a Ti35Zr28Nb alloy were found to enhance cell growth compared with the bare alloy [28]. Hierarchical micro-nanostructures were reported to improve osteointegration of titanium in in vivo animal experiments [27].

## 4. Conclusions

We propose a simple and viable two-step approach, anodic oxidation followed by chemical etching, to model the micro-nanoporosity of niobium oxide to promote osseointegration. The anodic oxidation of niobium was carried out in a Ca- and P-containing electrolyte. A sparking regime in pure galvanostatic or mixed galvanostatic/potentiostatic regime was investigated with 200 V and 250 V limiting potentials and a current density of 350 mA/cm^2^. At 250 V the oxide growth is very fast and within 10 s in pure galvanostatic regime a well-developed porous oxide layer is formed. Longer anodizing in mixed galvanostatic/potentiostatic regime leads to a thickening of the pore borders. The drawback of anodizing at 250 V is the formation of fractures in the oxide which can hinder the passivation properties of the layer. Lowering the potential to 200 V slows down the process but allows to avoid crack formation. In this case, longer anodizing in mixed regime is necessary to obtain a rather uniform porous oxide layer. The combined compositional and morphological analysis indicates long anodizing at 200 V as the best condition to grow porous, fracture-free oxide layers with Ca and P inclusions.

Post-anodizing etching was successfully employed to add nano roughness to the micro-sized pores of the oxide surface. Further studies are planned to investigate the effect of post-anodizing etching on the chemistry of the oxide surface as well.

In vitro tests using MG-63 osteosarcoma cells indicate that the hierarchical nano-microstructure helps cell spreading, promoting the generation of filopodia. The results indicate that niobium is a promising material for biocompatible implants and confirm the importance of nano/micro roughness in promoting osteointegration. To gain further insights into this aspect, future investigations will be performed combining high-resolution SEM analysis with fluorescence microscopy, to correlate cellular structure details (e.g., actin filaments or focal adhesion plaques) to surface nano/micro-morphology.

## Figures and Tables

**Table 1:** Roughness values (nm) calculated on 100 µm × 100 µm scan size areas.

Anodizing Conditions	Galvanostatic	60 s	300 s	30 min
**200 V**	480	570	550	660
**250 V**	710	820	620	-

## Supplementary Materials

The following are available online at https://www.mdpi.com/article/10.3390/ma15020473/s1. Figure S1: Current density/potential vs time curves, Figure S2: Polished vs anodized niobium.
