# Supplementary material for: A Two-Step Approach to Tune the Micro and Nanoscale Morphology of Porous Niobium Oxide to Promote Osteointegration
Source: Materials (Basel). 2022 Jan 8;15(2):473. doi: 10.3390/ma15020473 (PMC8778385; doi:10.3390/ma15020473)
Supplement: Supplementary file 1 [file materials-15-00473-s001.zip › materials-1532361-supplementary.pdf]

materials

Supporting Information

A Two-Step Approach to Tune the Micro and Nanoscale Morphology of Porous Niobium Oxide to Promote Osteointegration

Paolo Canepa [1], Giuseppe Firpo [2], Elena Gatta [3], Roberto Spotorno [4], Paolo Giannoni [5], Rodolfo Quarto [5,6], Maurizio Canepa [1,7], Ornella Cavalleri [1*]

[1] Dipartimento di Fisica and OPTMATLAB, Università di Genova, Via Dodecaneso 33, 16146 Genova, Italy; canepap@fisica.unige.it (P.C.); canepa@fisica.unige.it (M.C.)
[2] Dipartimento di Fisica and NANOMED, Università di Genova, Via Dodecaneso 33, 16146 Genova, Italy; firpo@fisica.unige.it
[3] Dipartimento di Fisica, Università di Genova, Via Dodecaneso 33, 16146 Genova, Italy; gatta@fisica.unige.it
[4] Dipartimento di Chimica e Chimica Industriale, Università di Genova, Via Dodecaneso 31, 16146 Genova, Italy; roberto.spotorno@unige.it
[5] Dipartimento di Medicina Sperimentale, Università di Genova, Via Leon Battista Alberti 2, 16132 Genova, Italy; paolo.giannoni@unige.it (P.G.); rodolfo.quarto@unige.it (R.Q.)
[6] U.O. Oncologia Cellulare, IRCCS Ospedale Policlinico San Martino, 16132 Genova, Italy
[7] INFN, Sezione di Genova, Via Dodecaneso 33, 16146 Genova, Italy
* Correspondence: cavalleri@fisica.unige.it

Supplementary materials

Citation: Canepa, P.; Firpo, G.; Gatta, E.; Spotorno, R.; Giannoni, P.; Quarto, R.; Canepa, M.; Cavalleri, O. A Two-Step Approach to Tune the Micro and Nanoscale Morphology of Porous Niobium Oxide to Promote Osteointegration. *Materials* **2022**, *15*, 473. https://doi.org/10.3390/ma15020473

Academic Editor: Swarup Roy

Received: 14 December 2021
Accepted: 5 January 2022
Published: 8 January 2022

Publisher's Note: MDPI stays neutral with regard to jurisdictional claims in published maps and institutional affiliations.

Copyright: © 2022 by the authors. Licensee MDPI, Basel, Switzerland. This article is an open access article distributed under the terms and conditions of the Creative Commons Attribution (CC BY) license (https://creativecommons.org/licenses/by/4.0/).

S1. Current density/Potential vs time curves

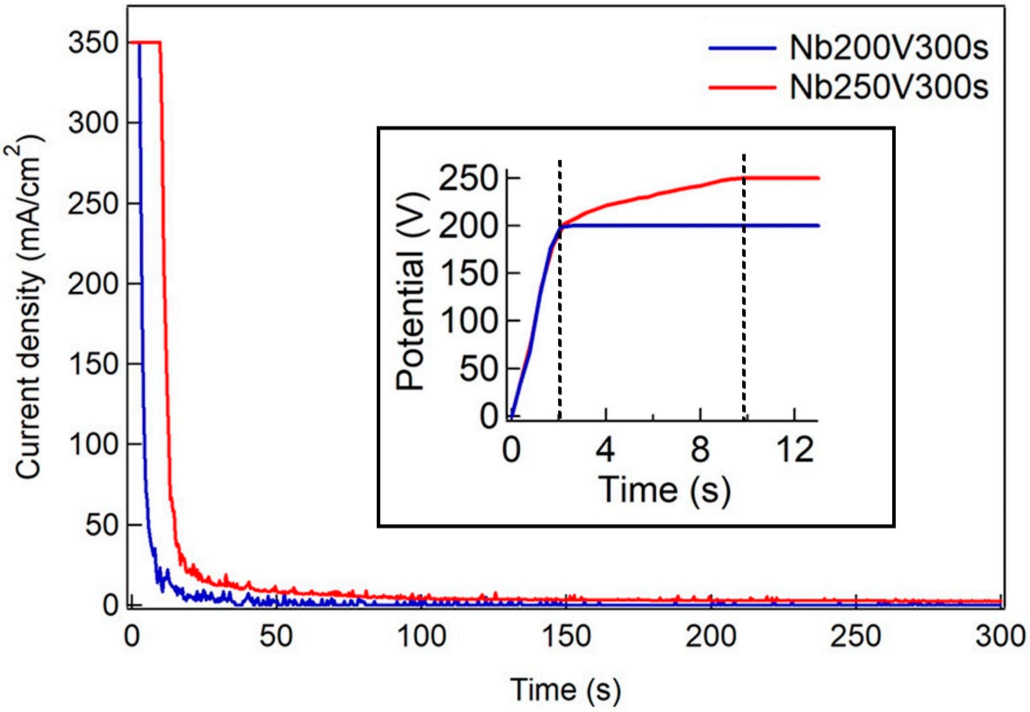

Figure S1. Typical current density vs time curves for Nb200V300s (blue line) and Nb250V300s (red line) samples. The inset reports the corresponding potential vs time curves.

Figure S1 reports the current density vs time curves for a typical anodizing of Nb200V300s (blue line) and Nb250V300s (red line) samples. After few seconds in galvanostatic regime with a constant current density fixed at 350 mA/cm^2, the current density quickly decreases asymptotically to zero. At the same time, the potential (reported in the figure inset) increases to the limiting value and remains stable until the end of the process. The time needed to reach the potentiotatic/galvanostatic transition depends on the chosen limiting potential, ~2 s for anodizing at 200V and ~10 s for anodizing at 250V (as indicated by vertical dashed lines).

S2. Polished vs anodized niobium

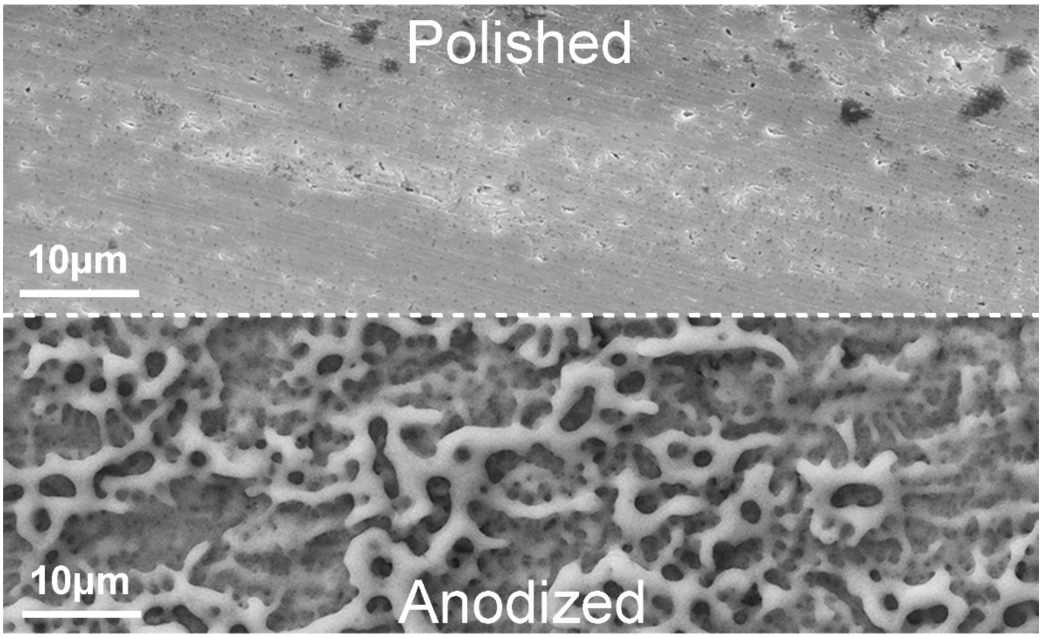

Figure S2. SEM images of polished (top) and Nb200V300s (bottom) niobium.

Figure S2 reports typical SEM images of a polished niobium sheet before anodizing (top part of the figure) and after anodizing (bottom part of the figure). The anodized sample is a Nb200V300s Nb sample. As can be inferred from the figure, anodizing heavily modifies the surface morphology.
